# Enhanced Disease Segmentation in Pear Leaves via Edge-Aware Multi-Scale Attention Network

**DOI:** 10.3390/s25165058

**Published:** 2025-08-14

**Authors:** Xin Shu, Jie Ding, Wenyu Wang, Yuxuan Jiao, Yunzhi Wu

**Affiliations:** 1 School of Information and Artificial Intelligence, Anhui Agricultural University, Hefei 230036, China; 2 Anhui Beidou Precision Agriculture Information Engineering Research Center, Anhui Agricultural University, Hefei 230036, China; 22115860@stu.ahau.edu.cn (X.S.);

**Keywords:** deep learning, disease segmentation, multi-dimensional joint attention, edge feature extraction module, convolutional network

## Abstract

Accurate segmentation of pear leaf diseases is paramount for enhancing diagnostic precision and optimizing agricultural disease management. However, variations in disease color, texture, and morphology, coupled with changes in lighting conditions and gradual disease progression, pose significant challenges. To address these issues, we propose EBMA-Net, an edge-aware multi-scale network. EBMA-Net introduces a Multi-Dimensional Joint Attention Module (MDJA) that leverages atrous convolutions to capture lesion information at different scales, enhancing the model’s receptive field and multi-scale processing capabilities. An Edge Feature Extraction Branch (EFFB) is also designed to extract and integrate edge features, guiding the network’s focus toward edge information and reducing information redundancy. Experiments on a self-constructed pear leaf disease dataset demonstrate that EBMA-Net achieves a Mean Intersection over Union (MIoU) of 86.25%, Mean Pixel Accuracy (MPA) of 91.68%, and Dice coefficient of 92.43%, significantly outperforming comparison models. These results highlight EBMA-Net’s effectiveness in precise pear leaf disease segmentation under complex conditions.

## 1. Introduction

Pears are widely cultivated worldwide due to their rich nutritional value and strong adaptability, making them a popular fruit tree variety. In agricultural economics, pear cultivation provides significant economic benefits to farmers, forming an essential component of their income [1]. However, pear trees are susceptible to various diseases, particularly under conditions of high humidity, poor ventilation, or high planting density. If these diseases are not controlled in a timely manner, they can severely impact fruit yield and quality, and may even lead to plant death. Although farmers can estimate the extent and severity of diseases based on experience, this approach is labor-intensive and lacks accuracy. With the advancement of computer technology, image segmentation techniques have enabled precise delineation of diseased areas, thereby facilitating quantitative analysis and providing technical support for a more accurate assessment of disease severity.

Image segmentation techniques include traditional feature extraction-based methods and convolutional neural network (CNN)-based deep learning approaches. Traditional feature extraction methods rely on recognizing image features and partitioning regions, typically involving steps such as image preprocessing, feature selection, region segmentation, and edge detection [2,3,4]. These traditional approaches usually utilize manually crafted features such as color histograms, texture descriptors, and shape features, followed by classifiers like SVM or k-NN. While effective in controlled or simple environments, these methods often struggle with complex agricultural scenes where disease appearances vary widely due to lighting conditions, leaf occlusions, and background clutter. Furthermore, the reliance on manual feature design limits their adaptability and robustness across diverse datasets.

In contrast, CNN-based deep learning approaches train on large amounts of labeled data and can automatically extract hierarchical and discriminative disease features without handcrafted preprocessing. This end-to-end learning capability enables the model to better generalize across varying conditions and complex backgrounds. For example, Fu et al. [5] proposed an improved DeepLabv3+ network using the MobileNetV2 backbone, which has been successfully applied to pear leaf disease segmentation. Similarly, Rai and Pahuja [6] employed an attention residual U-Net model based on deep learning to achieve effective real-time segmentation and detection of rice diseases. These studies show that deep learning methods have achieved remarkable results in processing complex disease images; however, there remains room for improvement in capturing fine edge details.

Based on these observations, deep learning approaches are preferred for their superior feature learning capacity, adaptability, and performance in complex scenarios, which motivates our adoption of a CNN-based model for precise segmentation of pear leaf diseases.

The U-Net network achieves precise pixel-level segmentation by integrating deep and shallow features through the combination of downsampling and upsampling processes, making it highly effective in disease segmentation tasks. Its symmetrical encoder–decoder structure, along with skip connections, significantly enhances the ability to capture detailed information in diseased regions, which is why it is widely used in plant disease segmentation tasks [7]. However, the U-Net model also has limitations: its fixed-size convolutional kernels are insufficient for handling irregular disease shapes, and its segmentation accuracy in complex backgrounds is not ideal. Therefore, incorporating attention modules that combine global and local contextual features may enhance U-Net’s ability to recognize diseases in complex backgrounds. Further adding an edge feature extraction path branch can help improve the model’s capability to capture features in boundary regions, and the synergistic effects between different feature paths can further enhance the model’s robustness.

Currently, pear leaf disease segmentation faces two major challenges: first, the diversity in the color, texture, and morphology of diseases makes it difficult for models to capture different disease features; second, variations in lighting and gradual color changes in diseased areas often result in blurred edges, and the resolution reduction in the encoder–decoder structure further exacerbates the loss of edge information [8].

To address the difficulty of capturing diverse features, Y. Zhao et al. [9] based on the DoubleU-Net framework, which incorporates multi-level feature fusion and an atrous decoder to effectively capture and understand contextual information. Riu et al. [10] designed an enhanced Dual Attention module (EDAM) to improve model performance by learning global feature correlations and developed a two-class feature fusion module (2-class FFM) to improve the accuracy of structural edges. G. Liu et al. [11] designed a Cross Attention and Feature Exploration Network, which combines cross-attention modules with upsampling modules, resulting in a cross-attention decoder that establishes connections between high-level and low-level features. This approach more effectively preserves low-level feature information and enhances the ability to restore details. However, this method still falls short in capturing fine details. Additionally, Yuan et al. [12], inspired by the original scSE module [13], designed an advanced attention mechanism called D-scSE. This mechanism introduces dynamic weights and a diversified pooling strategy to provide a more adaptive balance between the importance of spatial and channel information. Nevertheless, the aforementioned methods still exhibit limitations in handling complex disease shapes and balancing features under varying lighting conditions, restricting their practicality and accuracy.

Enhancing edge details is crucial for improving model performance. In terms of edge detail recovery, Patil and Kannan [14] used deep learning and classical segmentation techniques to predict maize leaf disease by converting images to HSV color space, creating binary masks based on color thresholds to isolate the green regions of leaves, and using edge detection (Canny Edge Detector) to identify the edges of diseased areas, thereby supplementing edge information to some extent. J. Zhou et al. [15] proposed a cascade structure of dilated branches with truncated gradient flow, which could alleviate low-level confusion and improve the performance of edge detection. To fully capture edge features, Bui et al. [16] proposed a Multi-scale Edge Guided Attention Network (MEGANet), which integrates the EGA model during the decoding process and uses the Laplacian operator to retain high-frequency information, preserving edge integrity and enhancing weak boundary detection. However, the extracted edge features were not well integrated into the model. Z. Zhu et al. [17] designed a Sparse Dynamic Volume Network with Multi-level Edge Fusion (SDV-TUNet), proposing a Multi-level Edge Feature Fusion (MEFF) module during the skip connection stage to combine low-level detail features containing spatial edge information with high-level global spatial features rich in semantic information. This approach facilitates the propagation and perception of spatial edge information, thereby enhancing the model’s sensitivity to edges. J. Zhao et al. [18] proposed a Multi-Scale Edge Fusion Network (MSEF-Net), which incorporates an Edge Feature Fusion Module (EFFM) combining Sobel operator edge detection and an Edge Attention Module (EAM) to fuse multi-scale edge features, and applies threshold processing (set to 0.5) to remove noise and irrelevant edge information, thus enhancing multi-scale edge features in images. Although numerous studies have explored edge handling mechanisms, most methods merely integrate edge processing into the backbone network. While this partially supplements edge information, segmentation accuracy remains suboptimal when lesion edges are blurred or color variations are not significant.

In summary, existing solutions show limitations in effectively capturing and balancing the diverse features of different pear leaf diseases in complex backgrounds. Most current methods either focus primarily on global feature extraction or edge detection, but seldom integrate both in a synergistic manner. Furthermore, their ability to restore fine edge details, especially for diseases with prominent or irregular edges, remains inadequate, which restricts the accuracy of disease segmentation.

To address these shortcomings, we propose the EBMA-Net segmentation model, which differs from previous works by incorporating a dedicated edge feature extraction branch (EFFB) alongside the main network. This branch explicitly enhances the model’s sensitivity to edge information, allowing better preservation and discrimination of lesion boundaries. Additionally, EBMA-Net introduces a Multi-Dimensional Joint Attention Module (MDJA), which simultaneously integrates global and local contextual information and adaptively balances channel features. This design enables more effective feature fusion and disease differentiation than traditional attention mechanisms used in earlier models.

Moreover, unlike many studies relying solely on public datasets or limited disease categories, we built a comprehensive dataset covering multiple pear leaf diseases under varied lighting conditions and occlusions, providing a robust benchmark for model evaluation. The primary contributions of this research are outlined as follows:

1. We introduced an Edge Feature Extraction Branch (EFFB), which consists of an Edge Feature Extraction module (EFE) and a Bilateral Feature Aggregation module (BFA). This branch is designed to extract edge features and fuse them with the outputs of the main network, effectively addressing the limitations of a single architecture in edge information extraction and enhancing the model’s sensitivity to details in complex environments.
2. We designed a Multi-Dimensional Joint Attention Module (MDJA), which includes two consecutive modules: the Global Multi-Scale Spatial Attention module (GMSA) and the Adaptive Grouped Channel Attention module (AGC). This module integrates global and local features, deeply explores the intrinsic relationships between channels, and further enhances the model’s ability to capture features of different diseases.
3. We collected data on three types of diseases, covering images under various lighting conditions and with leaf occlusions in complex environments, to comprehensively evaluate the model’s generalization ability and robustness, ensuring stable performance in diverse real-world application scenarios.

The rest of this paper is structured as follows: Section 2 describes the construction of the dataset and the introduction of our model. Section 3 presents the experimental setup, the loss functions and evaluation metrics used by the model, various experimental results, and an analysis of these results. Finally, Section 4 is provided, along with prospects for future research. The dataset and partial implementation code are available at our GitHub repository: https://github.com/echo080105/EBMA-Net (accessed on 23 July 2025).

## 2. Materials and Methods

### 2.1. Datasets

#### 2.1.1. Image Collection

**Figure 1:**
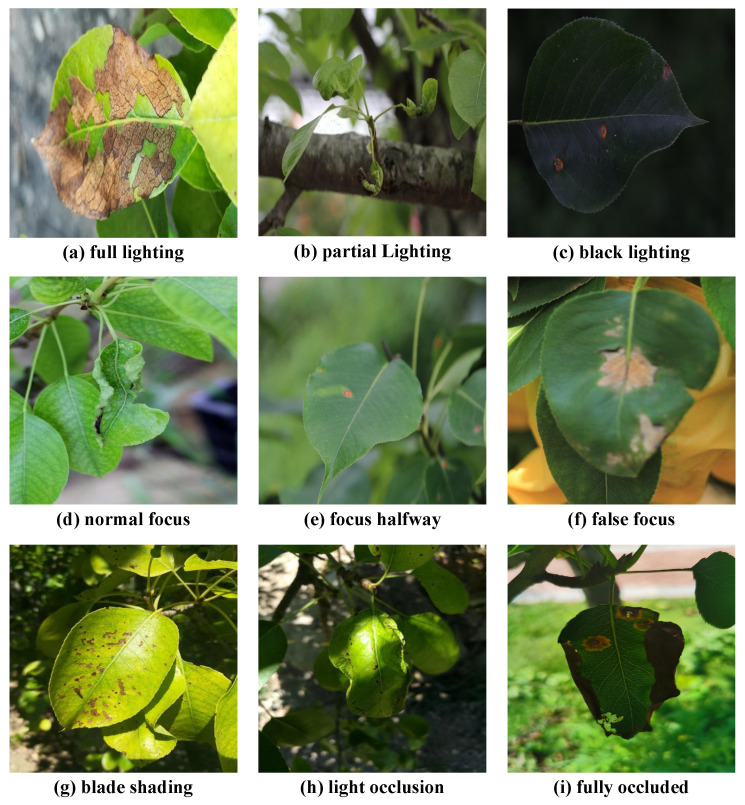
Different disease images under various conditions.

In this study, images of three types of diseases were collected: rust, brown spot, and leaf curl. The data mainly came from Dangshan, Suzhou, Anhui Province; Yingshang County, Fuyang City; and Tianmen Town, Tongling City, Anhui Province, China. Considering the impact of different lighting conditions, images of diseased leaves were taken at different times of the day from multiple angles. Additionally, to address the issue that many existing datasets deliberately focus on the diseased regions during collection—potentially reducing the model’s adaptability to disease recognition in real natural environments—we avoided intentionally focusing on diseased areas. Instead, we captured images containing the complete disease manifestation to better simulate how diseases appear in natural environments, thus enhancing the model’s generalization ability in real-world scenarios. All images were captured in outdoor environments, using a Canon 700D camera (Canon Inc., Hefei, China) and an Apple iPhone 15 Pro (Apple Inc., Hefei, China). An example of the captured samples is shown in Figure 1.

Due to differences in the resolution caused by the use of different devices, we standardized all image resolutions to 512 × 512 pixels and removed images that were poorly taken or of low quality. The final number of retained images was 1664.

#### 2.1.2. Dataset Construction

**Figure 2:**
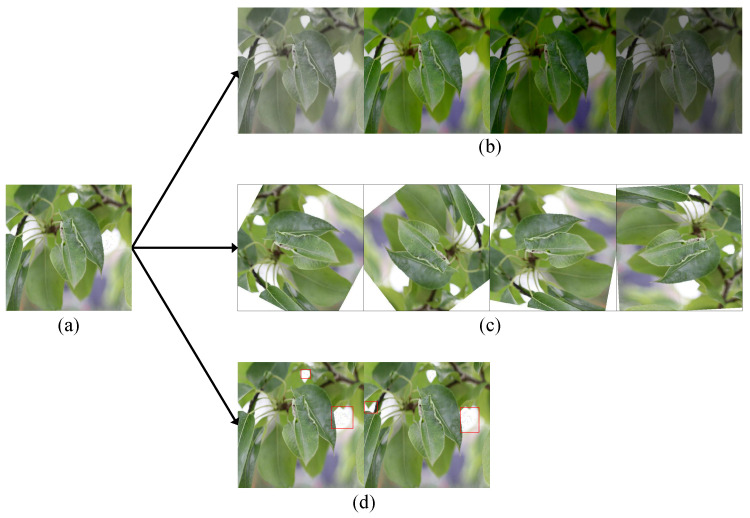
Data augmentation examples showing different conditions. (**a**) is original image. (**b**) is an enhanced image that has been adjusted for brightness and contrast, and processed by color transformation. (**c**) is an enhanced image after random rotation. (**d**) is an enhanced plot with Gaussian noise added.

We used the Labelme tool to perform simple and efficient manual annotation of disease regions. Each type of disease was independently annotated by a single trained annotator following a consistent and standardized labeling protocol to ensure annotation uniformity and minimize potential bias. To further enhance annotation quality and reliability, all labeled data underwent a second round of validation by a different annotator. In cases where notable discrepancies were observed between the two rounds, final decisions were made with the assistance of domain experts. This two-stage annotation process ensured high-quality labels across disease categories. Based on the finalized annotations, data augmentation was performed on two types of diseases, including random rotation, brightness and contrast adjustment, color transformation, and adding Gaussian noise to expand the dataset Figure 2), thereby enhancing the model’s generalization ability.

In addition, we selected relevant disease images from the public DiaMOS Plant Dataset [19] to supplement the initial dataset, avoiding the problem of uneven data distribution caused by a limited number of certain disease images. Detailed information about the samples in the self-constructed dataset and their annotations is shown in Table 1.

### 2.2. EBMA-Net Segmentation Architecture

**Figure 3:**
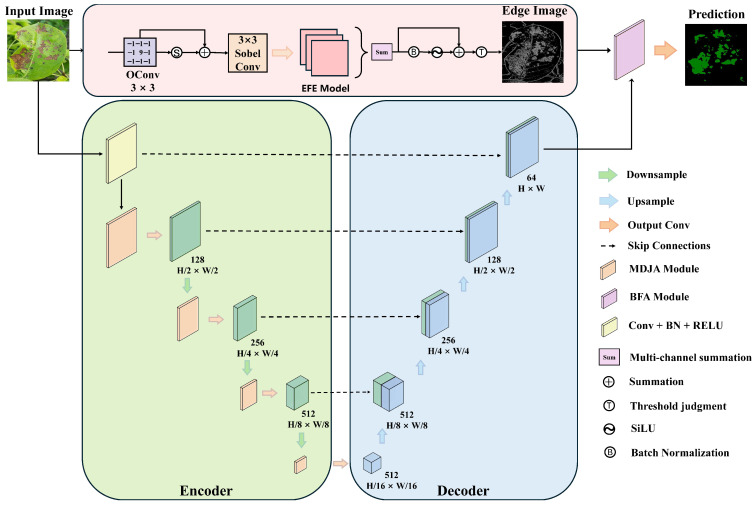
The proposed EBMA-Net architecture for pear leaf disease segmentation.

The proposed network architecture for pear leaf disease segmentation, EBMA-Net, is shown in Figure 3. The network mainly consists of the following two parts:

(1) Lightweight Edge Feature Extraction Branch: This branch extracts edge features from the original image through the EFE module, utilizing a self-constructed 3 × 3 convolution operator and the Sobel operator to enhance edge feature responses, thereby preserving edge information to the maximum extent. The extracted edge features are then input into the BFA module, where they are aggregated with the features from the main branch. By capturing both coarse and fine information from the edge and main features, this enhances the model’s consistent understanding of edge details and overall semantics. This branch not only provides rich edge information but also effectively reduces the interference of irrelevant features, significantly improving the segmentation accuracy of leaf disease regions and their edges.
(2) Main Branch with MDJA Module: This branch is based on the U-Net architecture and serves as the backbone structure of the entire network, leveraging classical upsampling and downsampling to fuse multi-level features. To simplify the network structure, we replaced the dual-layer convolution in U-Net with a single-layer convolution to reduce the number of parameters and improve computational efficiency while maintaining model performance. In addition, the MDJA module is introduced before each downsampling, enhancing the model’s feature perception capability and helping to accurately extract both unique and common features of diseases during downsampling, thereby further improving segmentation accuracy and robustness.

### 2.3. Multi-Dimensional Joint Attention Module (MDJA)

**Figure 4:**
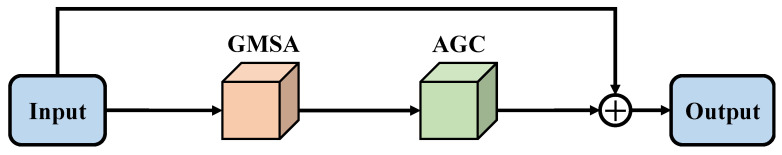
The structure of the Multi-Dimensional Joint Attention (MDJA) module.

To balance feature relationships among multiple diseases and effectively capture both common and unique features of various diseases under different environments, we designed the Multi-Dimensional Joint Attention (MDJA) module and applied it before each downsampling. This module helps the model dynamically adjust its focus, thereby enhancing both local detail and global pattern recognition abilities. It aids in separating diseased regions from background interference in complex backgrounds, significantly boosting the model’s expressive and discriminative capabilities, and enhancing its ability to capture features of different diseases. As a result, the model performs better in complex disease recognition tasks. The structure of the MDJA module is shown in Figure 4, and the overall process of the model is as follows:

(1) O=AGC(GMSA(X))+X

**Figure 5:**
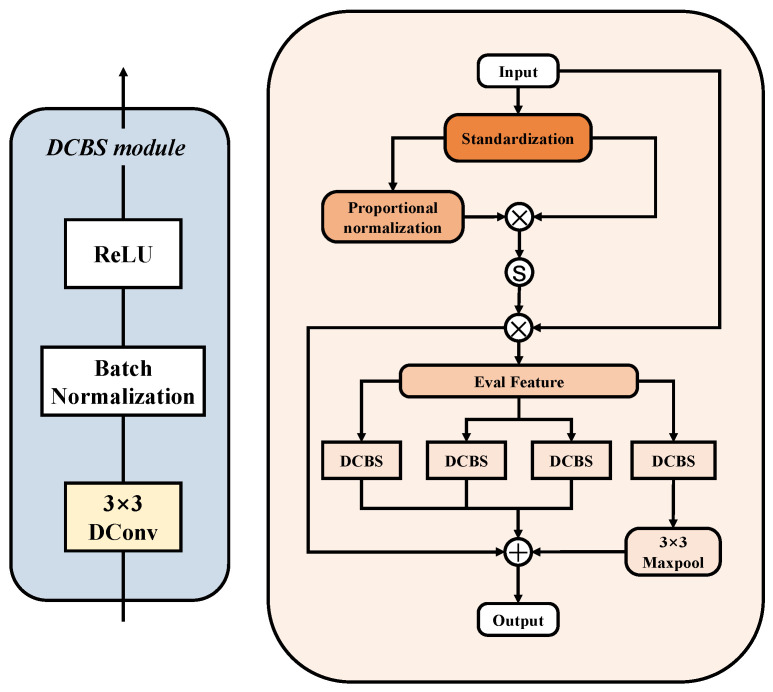
The structure of the GMSA module.

The GMSA module integrates information from the current stage and the previous stage through a strategy of global evaluation and multi-branch extraction, as shown in Figure 5. First, the input image $X\in R^{C\times H\times W}$ is scaled using a normalization formula to make the relative importance of each pixel clearer. Since this module is applied after each downsampling operation in the decoder, the spatial resolution H×W of the input varies across stages and may take values such as 512×512, 256×256, 128×128, 64×64, and 32×32. Then, the pixels are normalized, and a Sigmoid function is applied to obtain weight values (ranging from 0 to 1). These weight values are multiplied element-wise with the initial input features to obtain an enhanced global feature representation $F_{p}\in R^{C\times H\times W}$. The specific evaluation process is as follows:

(2) $$w_{ij}=\frac{x_{ij}-\bar{x}}{\sigma }$$

(3) $$F_{p}=\left\{F_{ij}^{p}\right\}=\sigma \left(\frac{w_{ij}}{\sum_{i=1}^{m}\sum_{j=1}^{n}w_{ij}}\times w_{ij}\right)\times x_{ij}$$

where i,j represent the position (i,j) in the image, and $x,\bar{x},\sigma$ are the pixel value at the corresponding position, the mean of all pixel values, and the standard deviation, respectively. σ represents the Sigmoid function.

A multi-branch structure is used to perform multi-scale feature extraction on $F_{p}$. Specifically, multiple parallel DCBS modules are employed to extract global information from the input features, combined with max-pooling convolution to capture salient features from different regions. In the DCBS module, dilated convolutions with different dilation rates enhance the model’s ability to capture multi-scale information, effectively expanding the receptive field without increasing the number of parameters. The designed parallel structure overcomes the loss of long-distance information correlation caused by the grid effect, while also avoiding detail loss due to incomplete pixel participation. The DCBS module is represented as follows:

(4) $$F_{DCBS}=ReLU(BN\left(DConv_{3\times 3}^{dilated=k}\left(F_{p}\right)\right))$$

where ReLU represents the ReLU activation function, BN represents the Batch Normalization layer, and *DConv* is the dilated convolution, where *k* represents the dilation rate (in the figure, from right to left are 1, 1, 3, 5).

This multi-scale feature extraction method effectively enhances the model’s ability to extract features of leaf occlusion and lesions of different sizes. However, multi-scale deep feature extraction also increases the difficulty of model training. To address this, we introduced a residual connection that merges the input features with the output of the multi-branch structure, improving the training speed and stability of the model. The final output of the GMSA module is $F_{S}\in R^{C\times H\times W}$.

**Figure 6:**
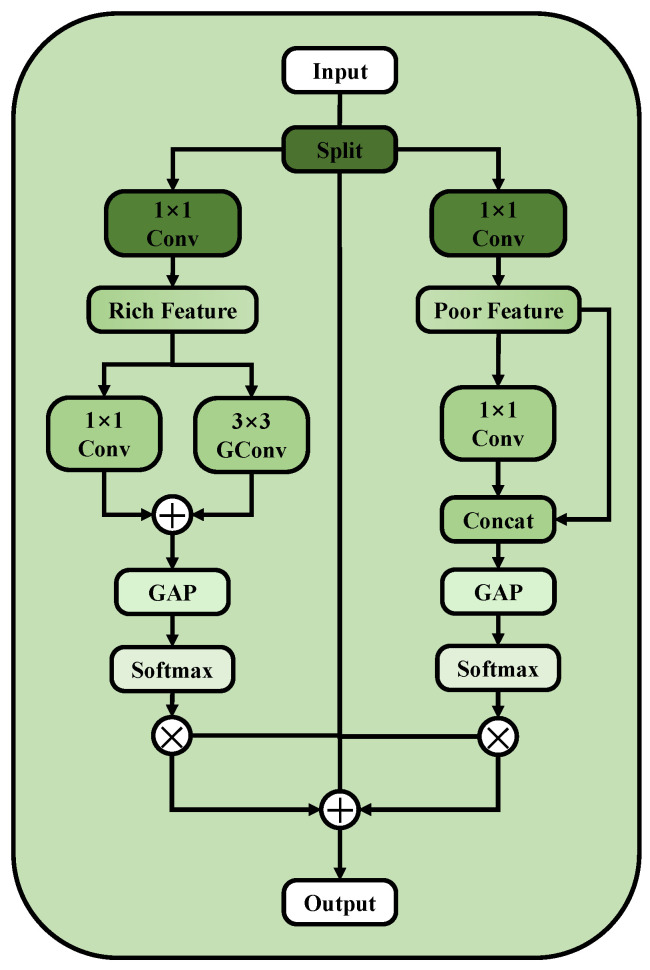
The structure of the AGC module.

The AGC module mainly adopts a dual-branch structure, as shown in Figure 6. First, through the channel split operation, $F_{S}$ is split into two features $F_{c1}\in R^{C_{1}\times H\times W}$ and $F_{c2}\in R^{C_{2}\times H\times W}$, each containing different levels of information richness, where the number of channels is αC and (1−α)C, respectively. This is intended to reduce interference between different disease features and enhance the independence and clarity of each type of disease feature. The channel splitting process can be represented as follows:

(5) $$F_{c1},F_{c2}=Conv_{1\times 1}\left(Split\left(F_{S}\right)\right)$$

where Split represents the channel split operation.

We introduce independent channel feature extraction methods for the two subspaces. For $F_{c1}$, which contains more information, we perform relatively complex feature extraction using parallel 3 × 3 grouped channel convolutions and 1 × 1 convolutions to deeply extract features. This combination allows different convolutions to share common information of different disease types, promoting information interaction between parallel input channels. Finally, the two output features are added element-wise to obtain feature $F_{co1}\in R^{C\times H\times W}$. For $F_{c2}$, which contains less information, we use a simple 1 × 1 convolution to briefly extract features at a lower computational cost, and then concatenate the extracted features with $F_{c2}$ along the channel dimension to obtain feature $F_{co2}\in R^{C\times H\times W}$. The specific feature extraction process can be represented by the following equations:

(6) $$F_{co1}=GConv_{3\times 3}\left(F_{c1}\right)+Conv_{1\times 1}\left(F_{c1}\right),\;F_{co2}=Concat\left(Conv_{1\times 1}\left(F_{c2}\right),F_{c2}\right)$$

where GConv represents a grouped convolution with 2 groups.

We perform global average pooling on $F_{co1}$ and $F_{co2}$, and apply the Softmax activation function to extract the weight of each channel. Then, the calculated channel attention weights are multiplied by the original feature maps to achieve dynamic adjustment of channel features. Subsequently, residual connections are used to further enhance feature representation, resulting in the final output feature map $F_{o}\in R^{C\times H\times W}$.

(7) $$F_{o}=Softmax\left(GAP\left(F_{co1}\right)\right)\times F_{s}+Softmax\left(GAP\left(F_{co2}\right)\right)\times F_{s}+F_{s}$$

In the final step of the MDJA module, we introduce a residual connection that adds the input feature map to the feature map processed by the attention mechanism. This preserves the original global features while enhancing the key features of each disease through the attention mechanism. This design not only improves the ability to distinguish and capture different disease features but also provides a comprehensive analysis and understanding of the disease images, helping the model to more accurately differentiate between diseased regions and complex backgrounds.

### 2.4. Edge Feature Extraction Branch (EFFB)

The Edge Feature Extraction Branch (EFFB) is a lightweight branch that we propose to extract edge features and fuse them with the output features of the backbone network. The goal is to enhance the model’s ability to recognize and capture edge information, thereby improving segmentation accuracy and robustness.

In the first stage of this branch, we use the EFE module (see flowchart in Figure 3 to extract edge features from the original image $X\in R^{C\times H\times W}$. Inspired by the Laplace operator [20], we designed a similar convolution kernel that highlights the edges in the image by calculating the differences between the central pixel and the surrounding pixels. In this design, the weight at the center of the convolution kernel is set to a positive value of 9, while the weights of the surrounding pixels are set to −1. After using this convolution kernel to enhance the edge features of the entire image, we apply the Sigmoid function to extract the corresponding weight for each pixel and multiply the generated spatial weight map with the initial features. The enhanced feature map $F_{e}\in R^{3\times H\times W}$ is then obtained through a residual connection. The convolution kernel we designed emphasizes the differences between neighboring pixels and the central pixel, allowing it to capture areas in the image with significant changes in pixel values, while producing relatively weaker responses in flat regions (i.e., areas with small pixel value changes), thereby improving the detection of boundaries in diseased regions. The enhancement formula is as follows:

(8) $$F_{e}=X+\sigma \left(OConv\left(X\right)\right)\times X$$

where OConv represents the custom-weight convolution with the convolution kernel weights specifically defined as follows:

$$\left[\begin{array}{ccc} -1 & -1 & -1\\ -1 & \;\;9 & -1\\ -1 & -1 & -1 \end{array}\right]$$

σ represents the Sigmoid function.

Next, we perform a per-channel convolution operation on $F_{e}$ based on the Sobel operator, allowing the edge features of each channel to be processed independently. This helps the model capture the edge features of the lesion spread direction. Then, the features from different channels are compressed into a single channel by adding them element-wise, effectively integrating multi-channel edge information to obtain the feature overlay map $F_{s}\in R^{1\times H\times W}$. This design allows the model to confine the disease edges within a certain range, thereby reducing incorrect feature extraction for diseases with blurred boundaries. The extraction process can be represented by the following equation:

(9) $$F_{s}=Sum\left(SiLu\left(BN\left(SConv\left(F_{e}\right)\right)\right)\right)$$

where SConv represents a per-channel convolution using the Sobel operator to extract edge features.

The feature map undergoes threshold processing, where parts below the threshold are set to zero and parts above the threshold are set to one, forming a binary saliency spatial weight map. In our implementation, a threshold value of 1 was empirically selected. Specifically, feature responses strongly related to edge regions typically exceeded 1, while irrelevant background features were mostly below this value. Therefore, this threshold effectively suppresses noise and preserves salient edge structures. Then, the Sigmoid function is applied for further smoothing to obtain the final saliency spatial weight map. This weight map is assigned as a weight to the input feature map, recalibrating the edge information to obtain an enriched edge feature map $F_{E}\in R^{1imesHimesW}$. Through this process, the model can reduce irrelevant information in complex backgrounds and focus more on the diseased areas in the leaves. The thresholding process is represented as follows:

(10) $$F_{E}=F_{s}+\sigma \left(Threshold\left(F_{s}\right)\right)\cdot F_{s}$$

where Threshold represents the thresholding process with a threshold value set to 1. σ represents the Sigmoid function.

**Figure 7:**
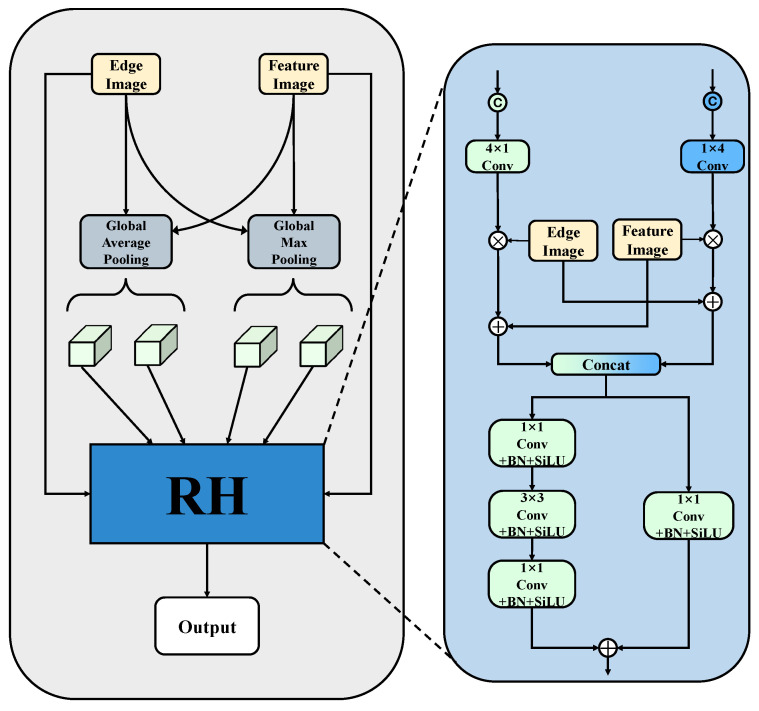
The structure of the BFA module.

The second stage of the branch, namely the BFA module, is shown in Figure 7. It aims to aggregate edge features into the backbone network, guiding the model to better focus on edge information. First, the outputs from different branches, $F_{E}$ and $F_{U}$ (the output features of the backbone network), undergo separate Global Average Pooling and Global Max Pooling operations to capture background region features and more prominent expression features. The extracted features are then concatenated along the spatial dimensions *H* and *W*, followed by adaptive size convolution processing. The Sigmoid function is then used to obtain the weights that emphasize features in different dimensions, resulting in weights $w_{H}$ and $w_{W}$. After obtaining the weights, the edge feature $F_{E}$ and backbone feature $F_{U}$ interact according to Equation (11), producing outputs $F_{E}^{R},F_{U}^{R}\in R^{C\times H\times W}$. These weighted feature maps mutually overlay features from both sources, effectively fusing information from different origins, ensuring completeness and complementarity of the information, and enhancing the model’s ability to parse complex scenes.

(11) $$F_{E}^{R},F_{U}^{R}=F_{E}\times w_{H}+F_{U},\;F_{U}\times w_{W}+F_{E}$$

In the output stage of the BFA module, a dual-path structure is used to learn the feature interaction relationships between different feature maps. We concatenate $F_{E}^{R}$ and $F_{U}^{R}$ along the channel dimension to form fused features, which are then fed into two path modules for processing. In the main path, the concatenated features pass through three successive convolution layers to leverage the advantages of consecutive convolutions for extracting local features of the disease. First, a 1 × 1 convolution is used for dimensionality reduction, followed by a 3 × 3 convolution for deeper feature extraction, and finally, a 1 × 1 convolution to restore the original dimensions. This dimensionality reduction reduces the model’s dependency on input feature dimensions and helps the model integrate global information. Additionally, the other branch passes through a 1 × 1 convolution, Batch Normalization (BN), and SiLU activation, and is merged with the main path input through element-wise addition to recover and enhance features that may have been weakened during the individual processing, thereby further improving the overall model performance. The specific calculation process is as follows:

(12) $$F_{R}=Conv_{1\times 1\to 3\times 3\to 1\times 1}\left(Concat\left(F_{E}^{R},F_{U}^{R}\right)\right)+Conv_{1\times 1}\left(Concat\left(F_{E}^{R},F_{U}^{R}\right)\right)$$

## 3. Results

### 3.1. Running Environment

All experiments were conducted based on the PyTorch framework in the same hardware and software environment. The specific experimental environment parameters are shown in Table 2, including hardware configuration, software versions, and experimental settings, to ensure the reproducibility and consistency of the experimental results.

To ensure the rationality of training, we randomly split the dataset into a 70% training set and a 30% test set. The training set was used for learning model parameters, while the test set was used to evaluate the model’s performance and verify its generalization ability on unseen data.

### 3.2. Loss Function

In this study, we adopted the Dice loss strategy. Dice loss focuses on the overlap between the predicted results and the true labels, which is particularly effective for the segmentation of small objects and can effectively alleviate class imbalance problems, especially when the background pixels significantly outnumber the disease pixels. The Dice loss function is specifically defined as follows:

(13) $$L_{Dice}=\frac{2\times TP_{i}}{2\times TP_{i}+FN_{i}+FP_{i}}$$

where $TP_{i}$ represents the True Positive for class *i*, indicating the number of pixels correctly predicted as belonging to class *i*. $FN_{i}$ represents the False Negative for class *i*, indicating the number of pixels that actually belong to class *i* but were not correctly predicted as such (missed detection). $FP_{i}$ represents the False Positive for class *i*, indicating the number of pixels incorrectly predicted as belonging to class *i* (false detection).

### 3.3. Model Evaluation Metrics

We used the following evaluation metrics: Mean Intersection over Union (MIoU), Mean Pixel Accuracy (MPA), Dice coefficient, and model parameters (Parameters). These metrics together provide a comprehensive quantitative basis for evaluating the model’s performance in image segmentation tasks.

MIoU calculates the average Intersection over Union (IoU) for each class, where IoU represents the ratio of the intersection area to the union area between the predicted and actual regions. This metric is used to measure the accuracy of the model for each class.

(14) $$MIoU=\frac{1}{n}\sum_{i=1}^{n}\frac{TP_{i}}{FN_{i}+FP_{i}+TP_{i}}$$

where *n* is the total number of segmentation classes, and $TP_{i}$, $FN_{i}$ represent the True Positive and False Negative for class *i*, respectively.

MPA calculates the average pixel accuracy of the model across all classes. For each class, pixel accuracy is defined as the ratio of correctly classified pixels to the total number of pixels for that class. This metric effectively reflects the model’s classification performance across different classes, providing an important reference for a comprehensive evaluation of image segmentation quality.

(15) $$MPA=\frac{1}{N}\sum_{i=1}^{N}\frac{TP_{i}}{TP_{i}+FN_{i}}$$

where *N* is the total number of segmentation classes, and $TP_{i}$, $FN_{i}$ represent the True Positive and False Negative for class *i*, respectively.

The Dice coefficient is a statistical tool used to measure the similarity between two samples. In the field of image segmentation, it is used to evaluate the spatial overlap between the predicted segmentation result and the ground truth.

Parameters refer to the total number of trainable parameters in the network model. This metric is related to spatial complexity and not only determines the complexity of the network but also affects the training speed and computational resource requirements of the model.

### 3.4. Effectiveness Analysis

#### 3.4.1. Effectiveness Analysis of MDJA Module

To validate the effectiveness of the proposed MDJA module, we used the EBMA-Net network without the EFFB module as the baseline. Keeping the basic architecture unchanged, we conducted replacement experiments on the MDJA module to explore its effectiveness within the network. The specific experimental results are shown in Table 3.

From the results in the table, it can be seen that the network using the MDJA module improves MIoU, MPA, and Dice coefficient by 1.99%, 0.9%, and 1.23%, respectively, compared to the network using the typical attention module CBAM. These data indicate that the proposed MDJA module effectively extracts multi-scale disease features, enhances feature representation, and captures the unique characteristics of different diseases. In comparison with experiments using only the SE module and the AGC module, the AGC module achieved a 1.19% improvement in the main metric MIoU, mainly due to the designed dual-channel independent feature extraction mechanism, which allows the model to more effectively capture unique feature information in each channel and reduce interference between channels, thereby improving sensitivity to diverse disease features. In the experiment using only the SE module, the addition of the GMSA module resulted in significant improvements across all metrics, especially in the main metric MIoU, with an increase of 2.83%, thanks to the GMSA module’s excellent spatial feature capture capability. Whether combined with the SE module or with our designed AGC module, the GMSA module significantly enhanced the model’s performance. Analyzing the data in the table, it can be concluded that the designed MDJA module outperforms other compared attention mechanisms in all performance metrics, fully demonstrating the effectiveness of the module.

#### 3.4.2. Effectiveness Analysis of EFFB Module

**Figure 8:**
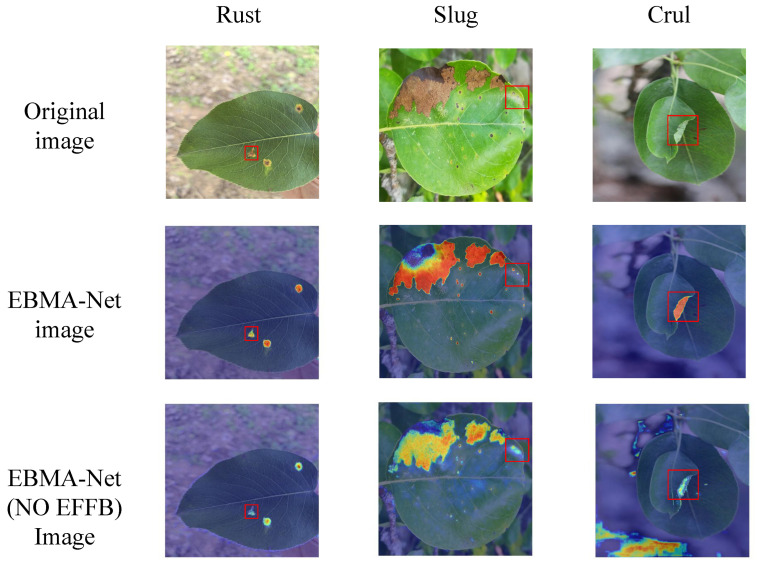
Heatmap visualization of attention points with and without the EFFB branch using Grad-CAM technology.

To intuitively demonstrate the effectiveness of our designed edge extraction branch (EFFB), we visualized the attention points of the EBMA-Net model with and without the EFFB branch based on Grad-CAM technology. The visualization heatmap results are shown in Figure 8.

When the edge feature extraction branch is added, the model effectively focuses its attention on the disease regions, reducing focus on irrelevant areas and thus avoiding performance loss. As shown in the figure above, in the segmentation of rust disease, the model not only focuses on the disease area but also accurately captures the disease boundary. For small spot brown spot disease, the model successfully concentrates attention on the disease area, significantly reducing ineffective attention to the surrounding area, thereby improving overall performance. When the model incorrectly identifies other elements or noise around leaf curl disease as disease, the edge extraction branch effectively controls false detection by supplementing edge detail information, helping the model more precisely determine the disease area and avoid interference from non-leaf region features. The introduction of the EFFB branch significantly enhances the model’s ability to focus on disease regions while improving its ability to perceive edge details, fully demonstrating the effectiveness of the edge feature extraction branch.

### 3.5. Impact of Different Input Scales

To evaluate the sensitivity of the model to changes in input image scales, we experimented with different input resolutions. These resolutions essentially cover all the settings used during model training, helping to analyze the impact of resolution changes on model performance. In this experiment, we normalized the computational load from 0 to the maximum value to intuitively show the growth trend between input resolution, computational load, and the main evaluation metric (MIoU). The specific experimental results are shown in Table 4.

**Figure 9:**
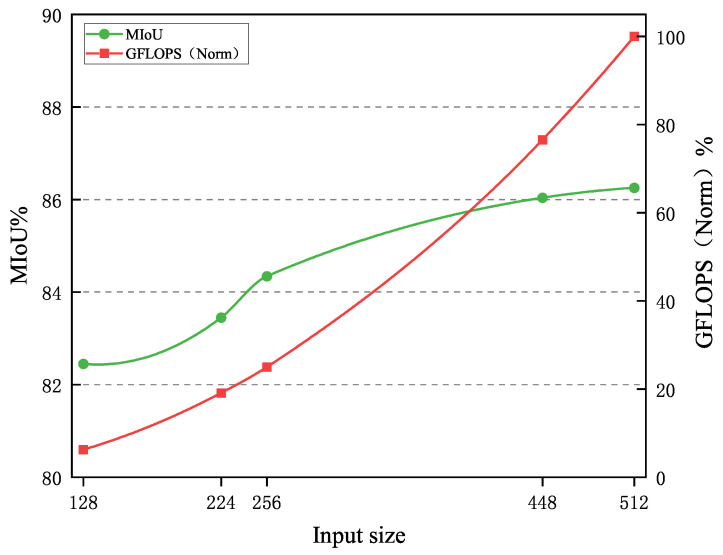
Relationship between MIoU and normalized computational load at different input scales.

The resolution of the input image directly affects the training efficiency and performance of the model. As the input scale increases, the model can capture more detailed features, but it also significantly impacts training speed. Figure 9 shows the relationship line chart between the main metric (MIoU) and normalized computational load at different input scales. As can be seen from Figure 9, as the resolution increases, the model’s performance gradually saturates, with limited improvement in evaluation metrics while training costs increase significantly. Therefore, all experiments in our design chose an input resolution of 512 × 512, which effectively balances computational cost and training speed while maintaining high model performance.

### 3.6. Model Structure Sensitivity Analysis

We also made some structural adjustments to the module, including changing the weighting method of feature interaction in the BFA module, replacing dilated convolutions with regular convolutions, and adjusting the position of the attention mechanism module to study the impact of these changes on model performance. These experiments provided important insights into understanding the internal working mechanisms of the model.

To explore the optimal fusion method in our BFA module, we designed comparative experiments involving different spatial feature weights. In the first experiment, we swapped the weights of the edge feature map and input feature map, using mismatched weights for horizontal and vertical features. In the second experiment, we averaged the two weights before performing weighted fusion. Through these comparative experiments, we aimed to determine which weight fusion strategy could better enhance model performance, thereby optimizing the effectiveness of feature fusion. The experimental results are shown in Table 5.

The comparative experimental results showed that both of these weighting methods performed worse than our designed weighted fusion strategy. This is because the two feature maps contain rich information in different directions, and the two weights respectively focus on vertical and horizontal features. Averaging the weights for fusion may weaken the representation of directional features, thereby affecting the fusion effect. Only through weighted fusion that is sensitive to the direction of the feature maps can the best effect be achieved, which is the weighting method adopted in our model.

Multi-convolution parallelism is a common strategy in the field of image processing. Compared to using a single large convolution, stacking multiple smaller convolutions usually yields better results. At the same time, without affecting the connectivity of the model, this method significantly reduces the number of parameters and computational complexity. Dilated convolutions allow small convolutions to achieve a larger receptive field, thereby enhancing the ability to recognize and segment small lesions, reaching a similar receptive range as large convolutions [21]. Therefore, reasonably setting the structure of the convolutions can effectively balance the relationship between model complexity and performance. We designed two comparative experiments to explore this: in the first, we set the dilation rate of all dilated convolutions in the GMSA module of the mixed attention mechanism to 1, that is, using regular convolutions of the same size; in the second, we replaced the dilated convolutions with large convolutions of the same receptive range. By comparing the experimental results of these two configurations, we can gain a deeper understanding of the role of dilated convolutions in capturing lesion details and their specific impact on model performance. The specific results are shown in Table 6.

From the comparative experimental results, the convolution mechanism designed in our model achieved 2.31%, 1.98%, and 0.0144 higher performance on various metrics compared to using regular convolutions. At the same time, with the parameter count being approximately half that of large convolutions with the same receptive field, better performance was achieved. This fully demonstrates the effectiveness and rationality of our designed convolution structure in balancing model complexity and performance.

Spatial attention mainly focuses on the positional information of key feature points in the image, while channel attention primarily focuses on the importance of different channels in the image [22]. To study the effectiveness of different attention structures and their impact on model performance, we designed three experiments in the following sequence: applying the AGC module first, followed by the GMSA module, running AGC and GMSA in parallel, and combining the effects of AGC and GMSA. These experiments aimed to explore the synergy between different attention mechanisms in the network and determine the optimal combination to further enhance the model’s performance. The specific results are shown in Table 7.

The comparative experimental results indicate that for leaf diseases, focusing on the location of the disease first and then extracting features of different diseases is the most effective structure. Specifically, we first use GMSA to capture the disease location, followed by AGC to extract features of different diseases in each channel. This strategy achieved the highest improvements in MIoU, MPA, and Dice metrics, with increases of 0.68%, 0.22%, and 0.042, respectively, outperforming the other two structural combinations. This fully demonstrates the effectiveness of the structure design of focusing on spatial attention first and then channel feature extraction in disease segmentation tasks.

### 3.7. Ablation Study

To explore the impact of our designed modules on the overall performance of the model and study the interaction between different modules, we conducted ablation experiments. Starting with the baseline network, we gradually added different modules to observe the specific contributions of each module to model performance. By comparing the experimental results under different module configurations (see Table 8), we systematically evaluated the role of each module in improving model performance as well as the synergistic effects between modules.

From the above table, it can be seen that the initial encoder–decoder network performed the worst across all evaluation metrics. This is primarily due to the lack of edge detail supplementation and insufficient feature extraction capabilities, which made it difficult to balance the feature extraction for different types of diseases, resulting in only coarse segmentation results. In contrast, after adding the GMSA module, the model’s spatial feature extraction ability was significantly improved. Its parallel convolution architecture effectively handled large disease areas, extracted more extensive features, and identified more disease characteristics. The introduction of the AGC module further enhanced the model’s ability to recognize and differentiate between different diseases, especially in cases where leaf curl disease and healthy leaves had similar color features, improving the model’s capacity to distinguish between similar features and thus reducing misclassification.

Comparing experiments with and without the EFFB module, it is evident that the EFFB module consistently enhanced the model’s performance in all scenarios. This improvement is attributed to its excellent edge feature extraction mechanism, which significantly increased the model’s sensitivity to disease edges, allowing it to effectively segment even when the edges were blurred. The complete EBMA-Net improved MIoU, MPA, and Dice metrics by 6.52%, 3.61%, and 0.0425, respectively, compared to the initial network, with a particularly notable improvement in the primary evaluation metric MIoU. Overall, the effective synergy of the EFFB, GMSA, and AGC modules resulted in outstanding performance in disease edge, spatial scale, and detail capture, fully validating the rationality and effectiveness of our designed structure.

**Figure 10:**
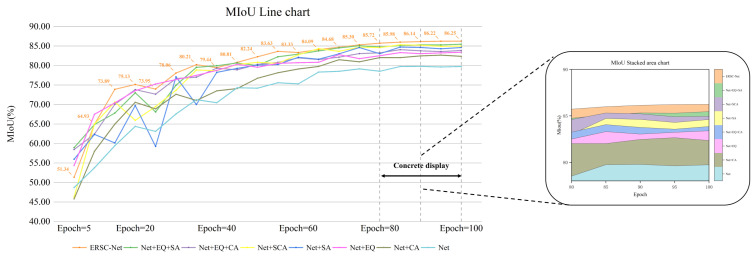
A graph of the MIoU of different models as a function of the number of trainings.

In addition, Figure 10 shows the changes in MIoU as the baseline model gradually adds each module. It can be observed that as the number of training epochs increases, the MIoU gradually converges. Although MIoU still shows a slight upward trend, the required number of training epochs increases significantly. This indicates that our model has been sufficiently trained on the training set, without showing signs of overfitting.

### 3.8. Performance on Different Disease Categories

To show that data imbalance did not negatively affect our model’s results, we examined how it performed on each disease category separately. The numbers in Table 9 reveal a fairly balanced accuracy across all classes. This suggests that our strategies—like data augmentation and using Dice loss—helped the model handle uneven sample sizes effectively.

### 3.9. Comparison Experiments

In the comparison experiments, we evaluated several state-of-the-art segmentation network models to analyze their performance in different scenarios. The comparison networks included classic U-shaped structures and their improved versions (UNet [7], UNet++ [23], I2UNet [24]), transformer-based networks (TransNet [25], SwinUNet [26]), encoder–decoder semantic segmentation networks (SegNet [27], FCN [28]), deep convolution-based networks (DeepLabv3 [29], DconnNet [30], LRASPP [31]), advanced residual networks (HRNet [32]), and networks that integrate multi-scale contextual information (PSPNet [33]). These networks cover the application of classical convolutional neural networks to modern attention mechanisms in semantic segmentation, aiming to comprehensively compare the performance of different methods in disease recognition tasks.

We conducted experimental comparisons between these networks and the proposed EBMA-Net. The results, summarized in Table 10, show each model’s performance on different evaluation metrics, including MIoU (Mean Intersection over Union), MPA (Mean Pixel Accuracy), Dice coefficient, model parameters (Parameters), and inference speed measured in FPS (Frames Per Second). These results provide an intuitive comparison of the strengths and weaknesses of each model in disease segmentation tasks and validate the effectiveness of our proposed EBMA-Net. The specific results are shown in Table 10.

**Figure 11:**
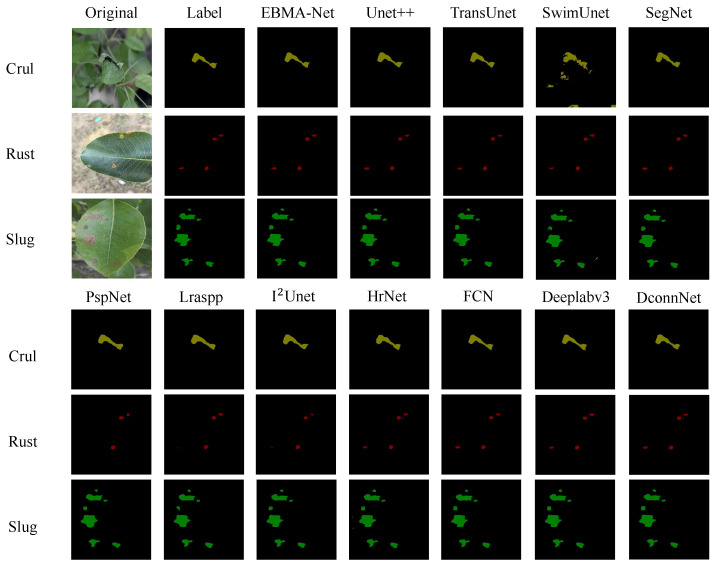
Comparison of the segmentation results of different models.

We performed disease segmentation predictions on all the compared networks, and the specific segmentation results are shown in Figure 11. In the figure, the prediction results of leaf curl disease, rust disease, and brown spot disease are marked in yellow, red, and green, respectively. From a visual perspective, it can be seen that our model effectively captured the shape features and color changes of the diseases, refined the edge segmentation effect, and fully processed the disease edges, accurately segmenting the detailed parts of the disease edges. Additionally, the model effectively suppressed false positives and false negatives, even constraining diseases with indistinct boundaries by limiting their edges to a certain range. Overall, our model achieved better results, proving its advancement and superiority.

**Figure 12:**
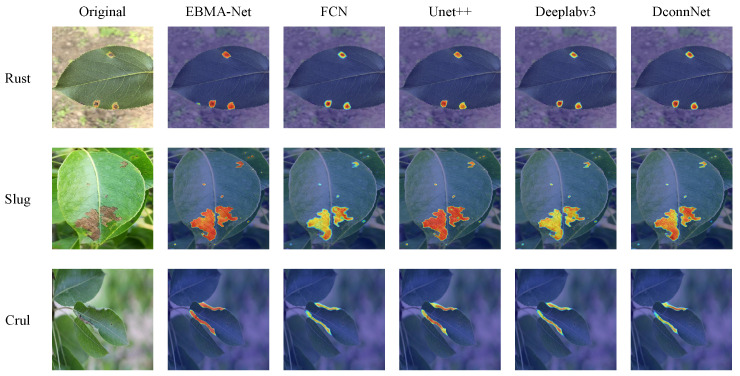
Grad-CAM visualization results of the top five networks in MIoU rankings.

To demonstrate the effectiveness of our model, we conducted a visual analysis of the attention maps for the top five networks in MIoU rankings using Grad-CAM technology. The specific results are shown in Figure 12. These visualization results clearly illustrate the feature-capturing capabilities of each model when focusing on disease regions, further validating the advantages of our model in both detail and global feature extraction.

With the integration of the EFFB branch, the model effectively focuses on the edges of various diseases, enhancing the delineation of subtle edges, particularly in rust disease. The prediction results indicate that the EFFB branch aids the network in concentrating on the affected regions, thereby mitigating performance loss during edge processing. The GMSA module exhibits outstanding spatial feature perception, capturing multi-scale features and effectively managing brown spot diseases of varying sizes. For leaf curl disease, characterized by subtle color features, the AGC module enhances the model’s ability to discern morphological differences and local structural damage, excelling in identifying and extracting features of leaf curl. Given the distinct activation patterns of different diseases across various channels, the AGC module captures valuable features more effectively, distinguishing between disease characteristics and achieving superior balanced performance in multi-disease segmentation tasks.

## 4. Conclusions

The proposed dual-branch EBMA-Net aims to address challenges such as the difficulty of extracting features from multiple diseases in complex environments, the difficulty of distinguishing between different diseases, and the indistinct edges of disease regions. Its goal is to achieve precise segmentation in challenging environments, providing technical support for agricultural disease prevention and control.

The designed multi-dimensional joint attention module integrates attention mechanisms to balance the ability to differentiate between various diseases, integrates both local and global features, and enhances the expression of key features while suppressing irrelevant ones, thereby strengthening the model’s feature extraction capabilities. The edge feature extraction branch employs various types of convolution operations to extract single-channel feature maps rich in edge information, effectively ignoring insignificant features and retaining critical edge details. Subsequently, the deep fusion of dual-branch output features significantly enhances the model’s focus on edge information.

Experimental validation on the self-built dataset demonstrates that EBMA-Net effectively extracts both edge and disease-specific features, achieving accurate disease segmentation. In comparison experiments, EBMA-Net outperformed other state-of-the-art methods, demonstrating its potential for practical agricultural applications. Although we have not yet collected datasets categorized by lighting and occlusion conditions, we recognize the importance of such analyses. Future work will include targeted data collection and classification evaluations to further validate and improve the model’s robustness under real-world complex scenarios.

Looking ahead, we plan to conduct further research into joint segmentation of multiple diseases and optimize the model architecture by incorporating depthwise separable convolutions to reduce the number of parameters and computational complexity. Meanwhile, although the proposed approach partially alleviates the interference caused by shadow occlusion, it still faces challenges under severe shadow conditions. We will explore shadow-invariant feature enhancement and domain adaptation strategies to further improve the model’s robustness.

## Figures and Tables

**Table 1:** Summary of dataset samples and their annotations.

Name	Representative Image	Annotating Images	Data	
			Original Data (1664)	Enhance Data (3100)
Rust	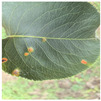	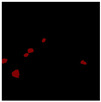	1046	1046
Curl	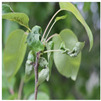	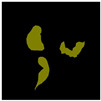	157	972
Slug	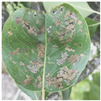	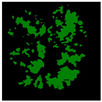	461	1082

**Table 2:** Experimental environment configuration.

**Hardware environment**	CPU	13th Gen Intel^®^ Core™ i9-13900K
	RAM	32 G
	GPU	NVIDIA GeForce RTX 4090 (24 G)
**Software environment**	OS	Windows 11 Professional Workstation Edition 64-bit
	CUDA Toolkit	11.3
	Python	3.8.13
	Torch vision	1.8.1
**Train environment**	Learning rate	0.0001
	Batch size	8
** **	Epoch	100
** **	Momentum	0.9
** **	Optimizer	adam
** **	Decay	cos

**Table 3:** Experimental results of MDJA module effectiveness.

Model	Evaluation Metrics			
	MIoU	MPA	Dice	Parameters
CBAM	83.23%	90.22%	90.56%	7.128 M
GMSA + SE	83.98%	92.22%	91.03%	19.673 M
SE	81.15%	90.41%	89.22%	7.127 M
AGC	82.34%	89.01%	89.97%	7.716 M
MDJA	85.22%	91.12%	91.79%	20.261 M

**Table 4:** Impact of different input scales on model performance.

Model	Evaluation Metrics			
	MIoU	MPA	Dice	GFLOPS(N)
128	82.45%	89.92%	90.03%	6.25%
224	83.45%	89.92%	90.67%	19.14%
256	84.34%	90.78%	91.22%	25.00%
448	86.04%	91.67%	92.30%	76.56%
512	86.25%	91.68%	92.43%	100.00%

**Table 5:** Comparison of different fusion methods in BFA module.

Model	Evaluation Metrics			
	MIoU	MPA	Dice	Parameters
Weight-swapping	85.86%	91.69%	92.19%	20.319 M
Weight-mean	85.81%	91.18%	92.16%	20.319 M
EBMA-Net	86.25%	91.68%	92.43%	20.319 M

**Table 6:** Comparison of dilated convolutions and large convolutions in GMSA module.

Model	Evaluation Metrics			
	MIoU	MPA	Dice	Parameters
Ordinary Conv	83.94%	89.70%	90.99%	20.319 M
Large Conv	86.12%	91.50%	92.35%	39.816 M
EBMA-Net	86.25%	91.68%	92.43%	20.319 M

**Table 7:** Comparison of different attention structures in the network.

Model	Evaluation Metrics			
	MIoU	MPA	Dice	Parameters
AGC + GMSA	85.98%	91.52%	92.26%	20.319 M
CAT (AGC, GMSA)	85.57%	91.46%	92.01%	21.016 M
ADD (AGC, GMSA)	85.83%	91.78%	92.17%	20.319 M
EBMA-Net	86.25%	91.68%	92.43%	20.319 M

**Table 8:** Ablation study results for different module configurations.

EFFB	MDJA		MIoU	MPA	Dice	Parameters
	GMSA	AGC				
			79.73%	88.52%	88.18%	7.084 M
	✔		84.60%	91.55%	91.40%	19.629 M
		✔	82.34%	89.01%	89.97%	7.716 M
	✔	✔	85.22%	91.12%	91.79%	20.261 M
✔			83.17%	90.14%	90.47%	7.141 M
✔	✔		85.46%	91.8%	91.93%	19.687 M
✔		✔	83.85%	90.74%	90.91%	7.773 M
✔	✔	✔	86.25%	91.68%	92.43%	20.319 M

**Table 9:** Performance comparison across different disease types.

Disease Type	Evaluation Metrics			
	MIoU	MPA	Precision	Recall
Crul	73.18%	84.42%	85.65%	84.34%
Slug	81.25%	88.77%	92.09%	88.12%
Rust	80.34%	88.63%	89.56%	88.92%

**Table 10:** Compare the results of segmented networks.

Model	Evaluation Metrics				
	MIoU	MPA	Dice	Parameters	FPS
Unet++	83.13%	89.22%	90.48%	26.905 M	62.21 FPS
TransNet	77.43%	85.33%	86.58%	66.815 M	71.16 FPS
SwinUnet	63.57%	82.96%	73.60%	41.342 M	56.91 FPS
SegNet	79.49%	87.16%	88.07%	29.445 M	40.93 FPS
PspNet	75.88%	85.70%	85.58%	49.06 M	137.21 FPS
Lraspp	79.74%	87.13%	88.21%	3.21 M	126.03 FPS
I^2^UNet	78.59%	85.03%	87.38%	7.03 M	49.11 FPS
HrNet	75.88%	86.15%	85.41%	29.35 M	25.91 FPS
FCN	84.36%	90.29%	91.27%	32.94 M	81.97 FPS
Deeplabv3	81.72%	91.33%	89.59%	39.63 M	72.79 FPS
DconnNet	80.28%	88.96%	88.65%	80.28 M	53.73 FPS
Unet	79.73%	88.52%	88.18%	7.084 M	29.73 FPS
EBMA-Net	86.25%	91.68%	92.43%	20.319 M	55.33 FPS

## Data Availability

All images used in this study were collected in real-world agricultural settings without involving any human subjects. The majority of the images were taken in experimental orchards managed by our laboratory, and the data collection was conducted with full permission. The dataset can be made available upon reasonable request.
